# Potential Utility of Sodium Selenate as an Adjunct to Metformin in Treating Type II Diabetes Mellitus in Rats: A Perspective on Protein Tyrosine Phosphatase

**DOI:** 10.1155/2013/231378

**Published:** 2013-09-11

**Authors:** Rania M. Salama, Mona F. Schaalan, Alaaeldin A. Elkoussi, Amani E. Khalifa

**Affiliations:** ^1^Pharmacology Department, Faculty of Pharmacy, Misr International University (MIU), KM 28 Cairo-Ismailia Road (Ahmed Orabi District), Cairo, Egypt; ^2^Biochemistry Department, Faculty of Pharmacy, Misr International University (MIU), Cairo, Egypt; ^3^Pharmacology Department, Faculty of Medicine, Assiut University, Cairo, Egypt; ^4^Pharmacology Department, Faculty of Pharmacy, Ain Shams University, Cairo, Egypt

## Abstract

Metformin is widely regarded as the standard first-line antidiabetic agent, in terms of efficacy and safety profiles. However, in most patients with type II diabetes mellitus (T2DM), it was found that metformin alone is not enough to adequately control hyperglycemia. Thus, we designed this study with the aim to investigate the effect of sodium selenate, a protein tyrosine phosphatase (PTP) inhibitor, individually and as an adjunct to metformin, on a rat model that simulates the metabolic characteristics of human T2DM. T2DM model was achieved by feeding the rats with high-fat, high-fructose diet (HFFD) for 8 weeks followed by a low dose of streptozotocin (STZ) (35 mg/kg/day, i.p.). Changes in serum glucose, insulin, adiponectin, homeostasis model assessment of insulin resistance (HOMA-IR) index, and the lipid profile were assessed. In addition, the level of reduced glutathione (GSH) and the activity of PTP were determined in the liver. Results showed that the addition of sodium selenate to metformin was able to restore hepatic GSH back to normal levels. Also, this combination therapy corrected the altered serum total cholesterol (TC), triglycerides (TG), and adiponectin levels. In conclusion, additive therapeutic effect was recorded when sodium selenate was used as an adjunct to metformin.

## 1. Introduction

It is estimated that diabetes mellitus (DM) accounts approximately for 6.8% of all deaths worldwide [1]. Egypt is expected to be ranked among the world's top 10 in terms of the highest number of people with DM in 2030 (8.6 million), which is much higher than the previous estimates in 2010 (4.7 million) [2]. However, these projections calculated by experts are probably underestimated as they are based only on the expected demographic evolution and do not take into account the evolution of obesity in the near decades.

The central etiological factor in the development of type II diabetes mellitus (T2DM) is the resistance of fat, muscle, and liver to insulin [3]. However, high flux of fructose to the liver was found to disturb glucose uptake pathways and enhance the rate of de novo lipogenesis and triglycerides (TG) synthesis [4]. Taking into consideration that a modern western diet not only contains high level of fructose but is also rich in both fat and cholesterol, synergistic interactions among these nutrients can occur leading to a greater degree of insulin resistance and dyslipidemia. 

The biguanide metformin is widely regarded as the standard first-line anti-diabetic agent, in terms of efficacy and safety profiles. This antidiabetic effect owes to metformin's ability to suppress hepatic glucose production [5], enhance peripheral glucose uptake [6], and improve peripheral insulin sensitivity [7]. However, hyperglycemia usually becomes poorly controlled by time, implying the need of an adjunct therapy.

As an attempt to introduce a novel combination, the promising sodium selenate was our focused objective in this study. It was shown long time ago that sodium selenate was effective in reducing plasma glucose level; however, the mechanism of action of sodium selenate was not fully understood at that time [8]. In 1996, Becker and his associates proved the efficacy of sodium selenate in enhancing glucose homeostasis and partly reversing the expression of liver glycolytic and gluconeogenic enzymes in diabetic rats [9]. Recently, many studies have focused particularly on the cytoplasmic phosphatase PTP1B as an important antagonist or negative regulator of insulin signaling owing to its ability to dephosphorylate the insulin receptor substrates (IRSs) 1 and 2 as well as the intracellular *β* subunit of the insulin receptor [10]. Interestingly; it was found that supranutritional sodium selenate doses can influence PTPs, involved in insulin-resistant DM, which in turn can be assumed as being responsible for the changes in intermediary metabolism such as gluconeogenesis and lipid metabolism [11]. 

However, the effect of sodium selenate on insulin resistance needs more attention which has tempted us to evaluate the possible increased efficacy of metformin after its concurrent administration with sodium selenate for 5 weeks, on a rat model that simulates the natural pathway and metabolic characteristics of human T2DM. This was assessed by detecting the changes in interrupted glucose metabolism via estimation of serum glucose, insulin, the adipocytokine “adiponectin,” homeostasis model assessment of insulin resistance (HOMA-IR) index, and lipid profile. In addition, the level of reduced glutathione (GSH) and the activity of PTP were determined in the liver.

## 2. Materials and Methods

### 2.1. Drugs and Chemicals

Sodium selenate, streptozotocin (STZ), and standard GSH were purchased from Sigma-Aldrich Chemical Company, USA. The feeding ingredients, such as casein, lard, and cellulose, were obtained from commercial sources and were of analytical grades. Fructose was purchased from Safety Misr Co., Egypt. Metformin hydrochloride (Glucophage) was purchased from Merck, USA.

### 2.2. Animals

Adult male Wistar rats weighing 100–120 g (National Research Center Laboratory, Cairo, Egypt) were housed in the animal facility of Faculty of Pharmacy, Misr International University, in standard polypropylene cages (four rats per cage). Prior to the dietary manipulation, they were fed normal pellet diet (NPD) (EL-Nasr Chemical Co., Cairo, Egypt) and permitted a free access to tap water. Rats were kept under standard conditions of temperature (22 ± 2°C) and relative humidity (55 ± 5%) with 12-light/12-dark cycles. Experimental design and animal handling were according to the guidelines of the Ethical Committee of the Faculty of Pharmacy, Ain Shams University, for Animal Use.

### 2.3. Dietary Model of Insulin Resistant Hyperglycemia (T2DM)

 Sixty rats were divided into two dietary regimen groups that lasted for a period of 8 weeks. Twelve rats were fed NPD (3.15 kcal/g; fat (5%), protein (21%), carbohydrate as starch (60%), fibers (3%) and vitamins and minerals (1%)) and this group served as normal control. Forty-eight rats were placed on a special high-fat, high-fructose diet (HFFD) to induce insulin resistance; the formula was obtained from Harlan laboratories (Teklad Diet TD.03293) (4.1 kcal/g; fructose (60%), lard (10%), casein (20.7%), cellulose (4.2%), mineral mix (3.5%), vitamin mix (1%), calcium carbonate (0.3%) and DL-methionine (0.3%)). Afterwards, hyperglycemia and overt diabetes were induced by an intraperitoneal (i.p.) injection of a single subdiabetogenic dose of freshly prepared STZ (35 mg/kg) [12] in citrate buffer (0.09 M, pH 4.8) after an overnight fasting. Normal control rats received i.p. citrate buffer only.

 To overcome the hypoglycemia which follows STZ, during the first 24 hours after their injection; diabetic rats were given 5% glucose solution to drink instead of tap water. Animals were monitored by periodic estimation of body weight and biochemical testing for fasting serum glucose. Only animals with persistent blood glucose levels higher than 200 mg/dL for 7 days after STZ administration were considered diabetic/insulin resistant (DIR) and were continued to be used in the study and started to receive treatment.

### 2.4. Groups under Investigation

One week after the STZ injection, rats that fulfilled the aforementioned criteria were randomly divided into 5 different groups, each of 12 rats as follows: Group 1 served as normal control rats, was fed NPD (3.15 kcal/g), and received single dose of citrate buffer (0.09 M, pH 4.8) alone i.p. Group 2 served as DIR rats. Group 3 served as DIR rats that received metformin (250 mg/kg/day; p.o.) dissolved in water [13]. Group 4 served as DIR rats that received sodium selenate (1.89 mg/kg/day; i.p.) dissolved in water [14, 15]. Group 5 served as DIR rats that received metformin (250 mg/kg/day; p.o.) plus sodium selenate (1.89 mg/kg/day; i.p.).

Groups 3, 4, and 5 continued the treatment for 5 weeks while being maintained on the same HFFD. The last dose of any treatment was given 24 hours before sacrificing the rats which fasted 14 hours before the time of sacrifice and blood samples were withdrawn, to minimize feeding-induced variations in lipid pattern and to measure fasting blood glucose level. 

### 2.5. Oral Glucose Tolerance Test (OGTT)

All groups were subject to an oral glucose tolerance test (OGTT) after 8 hours of fasting, during which animals were given an oral dose of aqueous glucose solution (2 g/kg) using oral gavage and blood samples were withdrawn at 0, 15, 30, 60, 90, and 120 minutes to evaluate the resulting glucose concentrations. Glucose was measured using Accucheck Compact (Roche Diagnostics, Almere, Netherlands).

### 2.6. Serum Separation

Blood was withdrawn from the retroorbital plexus of ether-anesthetized animals and centrifuged (3000 rpm, 4°C, 30 min) for separation of serum that was analyzed for glucose, insulin, free fatty acids (FFA), total cholesterol (TC), TG, and the adipocyte-secreted adiponectin. 

The glucose area under the curve (AUC) was calculated according to the following equation [16]:

(1) AUC=0.25(fasting)+0.5(12 hr  value)+0.75(1 hr  value)+0.5(2 hr  value).

Serum glucose was determined colorimetrically according to the glucose oxidase/peroxidase method [17], using a Stanbio Laboratories kit, USA. Sandwich type immunoassay technique was adopted to determine insulin content using an ELISA kit obtained from ALPCO Diagnostics, USA [18]. 

The HOMA-IR index was calculated according to the following equation [19]:

(2) HOMA-IR=Fasting  serum  glucose(mmol/L)×Fasting  serum  insulin(mIU/L)22.5.

Serum TC was determined enzymatically according to the cholesterol oxidase/4-aminophenazone method [20], using a Stanbio Laboratories kit, USA. Serum TG was determined according to the glucose oxidase/glycerylphosphate oxidase method [21], using a Stanbio Laboratories kit, USA. Serum FFA was determined colorimetrically according to the method of enzymatic conversion to acetyl-CoA and subsequently to H_2_O_2_ [22], using a kit obtained from BioAssay Systems, USA.

The adipocytokine, adiponectin, was measured using enzyme-linked immunosorbent assay (ELISA) kit obtained from Chemicon International, USA, which employs the quantitative two-step sandwich enzyme immunoassay technique [23]. 

### 2.7. Preparation of Liver Tissue Homogenate

Immediately after sacrificing the rats, dissection was done for isolation of the liver. Liver tissues (0.5 g) were excised and washed twice with phosphate buffered saline, dried between two filter papers, then homogenized in 5 mL phosphate buffered saline (10% w/v) using glass-Teflon Potter-Elvejhem device, divided into aliquots, and frozen at −70°C until assayed. For PTP assay; the concentration was calculated per mg protein where the protein content in each aliquot was assayed using Folin phenol reagent [24].

Liver content of GSH was determined depending on the fact that both protein and nonprotein SH-groups react with Ellman's reagent [5,5′-dithiobis-2-nitrobenzoic acid] (DTNB) to form a stable yellow color of 2-Nitro-5-thiobenzoic acid, which can be measured colorimetrically [25]. 

Activity of hepatic PTP was determined using PTP ELISA assay kit obtained from Sigma, USA, according to a method based on the in vitro colorimetric determination of protein tyrosine phosphatase (PTP) activity [26].

### 2.8. Statistical Analysis

Results are expressed as means ± SEM of 8 animals, and differences between groups were tested for significance using analysis of variance (ANOVA), followed by LSD *post hoc* test. The level of statistical significance was taken at *P* < 0.05, *P* < 0.01, and *P* < 0.001. Statistical analysis of the experimental data was performed using the statistical package SPSS for Windows (version 13.0, USA) and the GraphPad Prism (version 5, Graphpad software Inc., USA). CalcuSyn (Version 2.0, USA) was used as the definitive analyzer of combined drug effects.

## 3. Results

**Figure 1:**
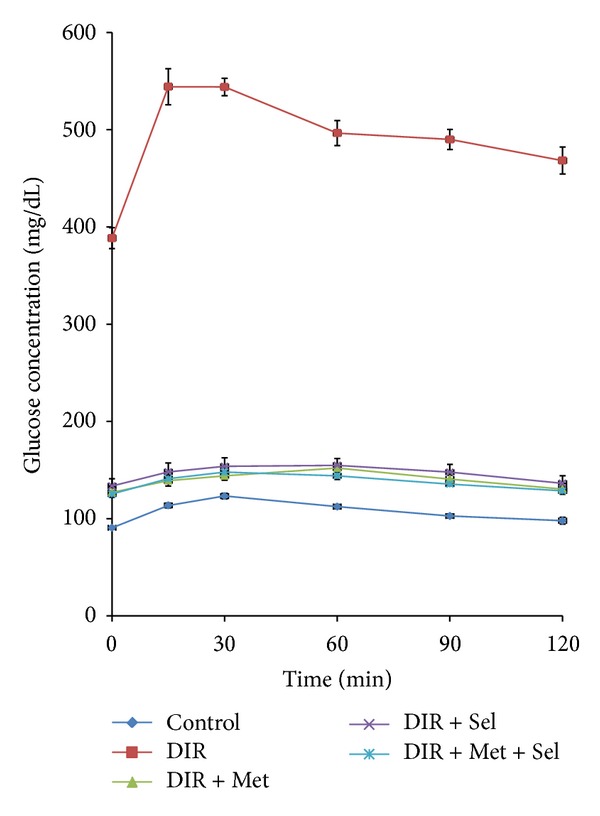
Glucose tolerance curve showing the effect of glucose (2 g/kg, p.o) in normal control, nontreated DIR, and treated rats with metformin, sodium selenate and their combination. Values represent the mean of 6 rats ± SEM (one-way ANOVA followed by LSD test). DIR: diabetic/insulin resistant, DIR + Met: diabetic/insulin resistant + metformin, DIR + Sel: diabetic/insulin resistant + sodium selenate, DIR + Met + Sel: diabetic/insulin resistant + metformin + sodium selenite.

The OGTT performed showed significant elevation in the glucose level in the DIR rats after oral administration of glucose (2 mg/kg, p.o), an effect that leveled off significantly after treatment with each of metformin, sodium selenite, and their combination (Figure 1).

**Figure 2:**
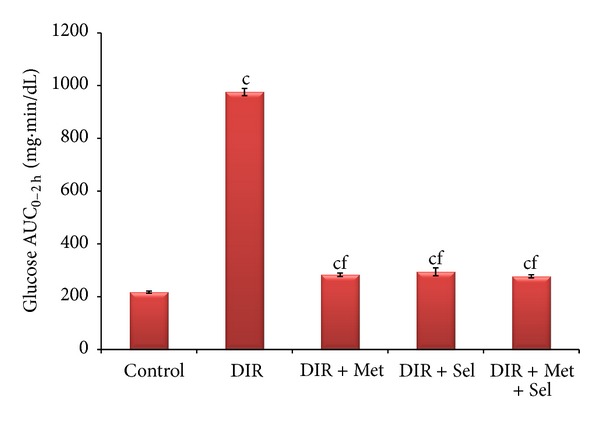
Changes in the glucose area under the curve (AUC) as derived from the OGTT for normal control, nontreated DIR and treated rats by metformin, sodium selenate and their combination at *P* < 0.05, *P* < 0.01, and *P* < 0.001. Values represent the mean of 6 rats ± SEM (one-way ANOVA followed by LSD test). DIR: diabetic/insulin resistant, DIR + Met: diabetic/insulin resistant + metformin, DIR + Sel: diabetic/insulin resistant + sodium selenate, DIR + Met + Sel: diabetic/insulin resistant + metformin + sodium selenite. ^(c)^ *P* < 0.001 compared to control group. ^(f)^ *P* < 0.001 compared to DIR group.

Data of OGTT was reflected on the glucose area AUC (Figure 2), showing a 4.5-fold increase in the DIR group compared to the control group, an effect that was significantly decreased in sodium selenate (DIR + Sel) (70%), metformin (DIR + Met) (71%), and metformin plus sodium selenate (DIR + Met + Sel) (71.5%) treated groups.

The DIR rats showed a 31% decline in their body weight, 3.7-fold increase in their fasting serum glucose, and 4-folds increase in HOMA-IR index, while serum insulin level showed 11% significant increase compared to the control group (Table 1). Metformin monotherapy showed significant increase in body weight compared to DIR rats (*P* < 0.001) and a significant decrease when compared to normal rats (*P* < 0.01). Sodium selenate monotherapy showed significant decline in body weight compared to the normal control rats (*P* < 0.001). As for the combination therapy of metformin and sodium selenate, it showed significant reduction in the body weight compared to metformin monotherapy (*P* < 0.01) and to normal control rats (*P* < 0.001).

Metformin monotherapy and the combination of metformin and sodium selenate were able to normalize the serum glucose at *P* < 0.01, while the sodium selenate monotherapy was able to normalize the serum glucose at *P* < 0.001. The metformin and sodium selenate combination treatment showed significant decrease in insulin and HOMA-IR index compared to the monotherapy with sodium selenate. However, this combination treatment showed significant increase in insulin level compared to monotherapy with metformin.

Results in Table 2 revealed a significant increase in serum level of TC (1.6 times), TG (2.5-folds), and FFA (1.8 times) in the DIR group compared to the control group. All treatments were able to normalize the serum TC level at *P* < 0.05. Monotherapy with metformin and sodium selenate reduced serum TC level by 38%. However, the combination of metformin and sodium selenate reduced serum TC level by 37%, when compared to the DIR group. 

As for serum TG, monotherapy with metformin and sodium selenate reduced its level compared to the DIR group by 43% and 51%, respectively, whereas, metformin and sodium selenate combined treatment reduced the serum TG level by 52%. This combination treatment was capable of restoring serum TG to normal level (*P* < 0.05), showing significant difference from metformin monotherapy (*P* < 0.05). However, the sodium selenate monotherapy was able to normalize serum TG level at *P* < 0.001. 

Monotherapy with metformin and sodium selenate reduced serum FFA level by 34% and 38%, respectively, whereas, metformin and sodium selenate combination treatment reduced the serum FFA level by 40%, which was the only treatment that was able to normalize the serum FFA level. Interestingly, this combination treatment also showed significant reduction in serum FFA level compared to the group receiving metformin only (*P* < 0.001).

As shown in Table 3, the HFFD/STZ exerted significant decline in the serum adiponectin level and the hepatic GSH content in the DIR group by 64% and 75%, respectively, whereas, hepatic PTP activity was significantly increased by 80% compared to the control group. All treatments failed to normalize the serum adiponectin level, showing no significant difference with the DIR group.

Metformin and sodium selenate monotherapy produced 3.4- and 3.5-folds increase in hepatic GSH, respectively, compared to the DIR group, where each of these 2 drugs managed to normalize the hepatic GSH at *P* < 0.01. However, the combination treatment of metformin and sodium selenate produced 3.7-folds increase in hepatic GSH compared to the DIR group, where this treatment was able to normalize the hepatic GSH level at *P* < 0.05.

Monotherapy with metformin and sodium selenate showed significant decrease in hepatic PTP activity by 14% and 39%, respectively, while the combination of both drugs showed a 39.5% significant decrease, compared to the DIR group. Treatment with sodium selenate, as single or combined therapy with metformin, was found to restore the hepatic PTP activity back to normal levels (*P* < 0.01). It was also noticed that this combination treatment showed significant reduction in the hepatic PTP activity compared to metformin monotherapy (*P* < 0.001).

Data in Table 4 shows the correlational analysis of glucose, HOMA-IR index, adiponectin, GSH, and PTP. Thus, HOMA-IR index is found to be positively correlated with serum glucose level (0.902, *P* < 0.01). As for hepatic GSH content, it is negatively correlated with serum glucose level (−0.843, *P* < 0.01) and HOMA-IR index (−0.717, *P* < 0.01) but positively correlated with serum adiponectin level (0.434, *P* < 0.01). Moreover, serum adiponectin level shows negative correlation with serum glucose level (−0.463, *P* < 0.01) and HOMA-IR index (−0.585, *P* < 0.01). As for hepatic PTP activity, it showed positive correlation with serum glucose level (0.702, *P* < 0.01) and HOMA-IR index (0.538, *P* < 0.01), while it showed negative correlation with serum adiponectin level (−0.515, *P* < 0.01) and hepatic GSH content (−0.756, *P* < 0.01).

## 4. Discussion

The simulation of T2DM was achieved by combining the feeding of HFFD, which produced insulin resistance, with a low dose of STZ treatment that caused the initial *β*-cell dysfunction and subsequently the frank hyperglycemia and mild hyperinsulinemia. These findings were associated with significant increase in HOMA-IR index, serum FFA, and hepatic PTP together with hypercholesterolemia and hypertriglyceridemia. On the other hand, remarkable reduction in serum adiponectin and hepatic GSH was observed, which supports the finding that oxidative stress in diabetes coexists with a decrease in antioxidant capacity such as GSH leading to an increase in the harmful effects of free radicals [27].

The hypoglycemic effect of metformin was shown in a recent study [28], which was further illustrated by OGTT done in another study [29], showing that plasma glucose excursion after oral glucose loading was significantly improved, and that glucose AUC was significantly decreased owing to extrapancreatic mechanisms such as the inhibition of hepatic glucose output in the liver and glucose absorption in the gut as well as enhancing peripheral glucose disposal. Confirming the current results, a recent study revealed a significant improvement in HOMA-IR index by metformin treatment indicating decreased insulin resistance [30]. Metformin produced significant elevation in insulin level which was supported by other studies [28, 31]. However, activation of adenosine-monophosphate activated protein kinase (AMPK) by metformin was proposedto be responsible for the markedlyreducedglucose-stimulatedinsulinrelease fromprimarypancreatic islets [32] and*β*-celllines[33].Thus, contradictory to our results, another study showed significant decrease in plasma insulin level after treatment with metformin [34]; this can be attributed to the different diet used to induce diabetes where hypercaloric diet was used leading to hyperinsulinemia in the diabetic rats. In addition, the study carried out by Ong and his group showed no significant change in serum insulin levels [35], which is probably caused by the large dose of STZ employed (65 mg/kg) leading to this severe decline in insulin levels and the unresponsiveness towards the large dose of metformin given (500 mg/kg). The lowering effect of metformin on the lipid profile was witnessed in other studies where metformin significantly decreased the serum level of TC, TG [31, 36], and FFA [30, 37]. Both studies done by Matafome and his associates as well as Ong and his coworkers showed opposite results in which there was no significant change in TC and TG [30, 35]. In the former study, T2DM was induced by a special HFD only, and rats received low dose of metformin (60 mg/kg/day) for 1 month. The study done by Hu and his group showed that metformin did not produce significant reduction in FFA levels [31], which could be attributed to the model used, where rats were fed high-fat and high-glucose chow followed by large single dose of STZ (60 mg/kg) and then treated with lower dose of metformin (200 mg/kg/day). Metformin monotherapy failed to improve the serum adiponectin level which is in agreement with the study carried out by Haddad and his group [34]. These results are different from the finding proposed by Metais and his associates, in which they postulated that metformin had a net stimulatory effect on both adiponectin receptors in muscle and a mild stimulatory action on liver AdipoR2 and white adipose tissue (WAT) AdipoR1, with an inhibitory effect on WAT AdipoR2 [38]. Other studies supported the beneficial effect of metformin on GSH level where metformin restored the GSH content in the kidney tissue in a dose-dependent manner [39], which was attributed to the ability of metformin to modulate the expression of several oxidative and proinflammatory genes at the transcriptional levels. Also, the study carried out by Behera and his group showed significant elevation in the liver, kidney and heart of diabetic rats treated with metformin [36]. On the contrary, the study done by Ong and his coworkers showed no significant change in both hepatic GSH and antioxidant enzymes in diabetic rats treated with metformin [35]. Interestingly, we recorded that metformin was able to significantly lower the level of hepatic PTP which is responsible for termination of insulin signal. This was supported by a study attributing this to a direct action, where metformin stimulated insulin signaling by increasing the tyrosine kinase activity of the *β*-subunit of insulin receptor, and an indirect action by inhibiting endogenous tyrosine phosphatases and purified human PTP1B that dephosphorylate and inhibit the insulin receptor kinase [40]. Also, the study done by Kannappan and Anuradha showed significant reduction in the level of PTP in liver homogenate by metformin in fructose-fed rats which reflected improved insulin signaling and sensitivity and thereby promoted the cellular actions of insulin [41]. 

The anti-hyperglycemic effect of sodium selenate was witnessed in a recent study, which suggested that this effect could be attributed to insulin-like actions of selenate that include stimulation of glucose uptake and regulation of metabolic processes such as glycolysis, gluconeogenesis and fatty acid synthesis [42]. A supporting evidence for the hypoglycemic effect of sodium selenate was shown in another study [43]; which attributed this effect to the reduction in protein sulfhydryl oxidation which may result in better activity of glucose transporters [44]. The improvement of glucose tolerance after a glucose challenge is in accordance with a previous study [45], in which diabetic mice treated with selenate showed recurrence of blood glucose concentration to the fasting level which was comparably as fast as in the initial status. Consequently, significant decline in HOMA-IR index by sodium selenate was witnessed in another study [46], which is in accordance with our results. However, the current results are contradictive to the past results that excluded any effect for sodium selenate on insulin receptor, attributing the glucose lowering effect to the translocation of glucose transporters from the intracellular compartment to the plasma membrane [8]. As for the lipid profile, previous studies showed that supranutritional selenate led to a significant decrease in plasma levels of TC [11] and TG [11, 42]. Furthermore, it was found that sodium selenate was able to normalize TC levels [42]. This efficacy of sodium selenate on sera TC and TG could be attributed to increased lipoprotein lipase activity leading to hydrolysis of TG into FFA followed by cellular uptake of FFA released [47]. It was shown in our study that sodium selenate decreased serum FFA level. This can be explained by the finding of Mueller and Pallauf, in which the supranutritional selenate doses increased the expression of peroxisome proliferator activated receptor-gamma (PPAR*γ*) [11]. The activation of PPAR*γ* was proven to repress the expression of genes that induce lipolysis and release of FFA, such as the *β*3-adrenergic receptor [48] and offensive cytokines like resistin, interleukin-6 (IL-6), and tumor necrosis factor-alpha (TNF-*α*) [49]. Further studies are needed to evaluate the effect of sodium selenate on serum adiponectin level, where our study showed no effect for sodium selenate over serum adiponectin level, although ligands that activate PPAR*γ* were proven to increase the expression of adiponectin [50]. In line with the current results, a previous study showed that administration of sodium selenate increased GSH level significantly [42]. This effect is exerted through the redox-active selenocysteine residue as an integral part of the selenoproteins glutathione peroxidases, where these selenoproteins are involved in the regulation of the antioxidative balance of tissues [51]. Supporting the current results, a previous study showed that the activity of cytosolic PTPs was reduced from 53.8% to 22.5% in the liver and skeletal muscle of selenate-treated mice [52]. Similar results were also obtained from the study of Mueller and Pallauf [11], in which selenate treatment inhibited the activity of PTPs as important antagonists of insulin signaling by about 50%, as compared to selenium-deficient and selenite-treated animals. 

In the current study we used a novel combination of metformin and sodium selenate. Serum glucose level was significantly reduced by this combination treatment illustrating additive effect; however, it could not restore glucose to normal values. This was further illustrated by the significant improvement in glucose tolerance as revealed by the OGTT response and the resulting significant decrease in glucose AUC illustrating additive effect produced by this combination. Interestingly, HOMA-IR index was significantly decreased proving the reduction of insulin resistance. At the same time, this combination treatment provided additive effect than using sodium selenate alone. As for serum insulin level, higher levels of insulin were obtained than after using metformin monotherapy, but lower values than after sodium selenate single therapy. This combination was able to successfully normalize serum TC and TG levels which were not achieved by each of these two drugs alone, illustrating the additive effect of administering these two drugs. As for serum FFA, using these two drugs together could not return it back to normal levels; however, it produced additive effect compared to metformin taken alone. Negative results were obtained involving serum adiponectin levels, where this combination treatment did not differ from using each drug alone proving that such drugs individually or in combination do not provide beneficial effect over serum adiponectin level. On the contrary, using these two drugs together provided additive effect concerning hepatic GSH which was successfully returned back to normal levels, an effect which was not achieved by single therapy of each of these two drugs. Finally, hepatic PTP levels after the combination therapy were found to be similar to sodium selenate monotherapy but significantly lower than metformin alone, illustrating the additive effect this combination produced compared to metformin monotherapy.

## 5. Conclusion

In this study we demonstrated the additive effect of sodium selenate when used as an adjunct to metformin, in reducing blood glucose level and other metabolic disturbances in T2DM rat model. Yet, further studies should be done to study the combined efficacy of sodium selenate with other antidiabetic drugs. Besides, assessment of other cytokines could serve as new indicators of antidiabetic efficacy, which may result in better and more efficient management of DM and its related complications.

## Figures and Tables

**Table 1:** Effect of daily administration of metformin (250 mg/kg; p.o.) [DIR + Met], sodium selenate (1.89 mg/kg; i.p.) [DIR + Sel], and metformin (250 mg/kg; p.o.) plus sodium selenate (5 mg/kg; i.p.) [DIR + Met + Sel] on body weight and serum content of glucose, insulin, and HOMA-IR index using DIR rats.

Groups	Parameter			
	Body weight (g)	Serum glucose (mg/dL)	Serum insulin (*μ*IU/mL)	HOMA-IR index*
Control	326.575 ± 7.77	91.493 ± 2.431	7.975 ± 0.491	1.805 ± 0.127
DIR	226 ± 12.202^c^	341.948 ± 28.385^c^	8.887 ± 0.216^a^	7.511 ± 0.699^c^
DIR + Met	289.5 ± 6.214^bf^	127.466 ± 1.666^af^	11.025 ± 0.220^cf^	3.473 ± 0.102^cf^
DIR + Sel	207.437 ± 10.821^c^	147.779 ± 11.384^bf^	15.162 ± 0.128^cf^	5.532 ± 0.431^cf^
DIR + Met + Sel	208.312 ± 2.864^ci^	124.466 ± 3.556^af^	12.365 ± 0.226^cflh^	3.799 ± 0.126^cfl^

Values represent the mean of 8 rats ± SEM (one-way ANOVA followed by LSD test).

^a^ *P* < 0.05, ^b^ *P* < 0.01, and ^c^ *P* < 0.001 compared to the control group. ^f^ *P* < 0.001 compared to DIR group. ^h^ *P* < 0.01, ^i^ *P* < 0.001 compared to DIR + Met group. ^l^ *P* < 0.001 compared to DIR + Sel group.

*HOMA-IR = Fasting serum glucose (mmol/L) × Fasting serum insulin (mIU/L)/22.5.

**Table 2:** Effect of daily administration of metformin (250 mg/kg) [DIR + Met; p.o.], sodium selenate (1.89 mg/kg; i.p.) [DIR + Sel], and metformin (250 mg/kg; p.o.) plus sodium selenate (1.89 mg/kg; i.p.) [DIR + Met + Sel] on serum content of TC, TG, and FFA using DIR rats.

Groups	Parameter		
	Serum content (mg/dL)		
	TC	TG	FFA
Control	64.814 ± 1.445	61.892 ± 3.357	16.400 ± 0.224
DIR	107.056 ± 3.565^c^	155.809 ± 2.236^c^	29.143 ± 0.395^c^
DIR + Met	66.089 ± 0.984^f^	88.193 ± 3.837^cf^	19.252 ± 0.161^cf^
DIR + Sel	66.381 ± 0.761^f^	76.821 ± 1.967^bf^	18.137 ± 0.287^cf^
DIR + Met + Sel	66.858 ± 1.066^f^	75.423 ± 2.171^f^	17.612 ± 0.377^bfi^

Values represent the mean of 8 rats ± SEM (one-way ANOVA followed by LSD test).

^b^ *P* < 0.01, ^c^ *P* < 0.001 compared to the control group. ^f^ *P* < 0.001 compared to DIR group. ^i^ *P* < 0.001 compared to DIR + Met group.

**Table 3:** Effect of daily administration of metformin (250 mg/kg; p.o.) [DIR + Met], sodium selenate (1.89 mg/kg; i.p.) [DIR + Sel], and metformin (250 mg/kg; p.o.) plus sodium selenate (1.89 mg/kg; i.p.) [DIR + Met + Sel] on serum content of adiponectin as well as hepatic content of GSH and activity of PTP using DIR rats.

Groups	Parameter		
	Serum adiponectin (ng/mL)	Liver GSH (mg/dL)	Liver PTP (pmol/mg protein)
Control	1.975 ± 0.224	33.576 ± 1.810	10.812 ± 0.169
DIR	0.712 ± 0.035^c^	8.283 ± 0.459^c^	19.518 ± 0.327^c^
DIR + Met	0.849 ± 0.083^c^	28.399 ± 1.464^af^	16.800 ± 0.250^cf^
DIR + Sel	0.940± 0.054^c^	28.840 ± 1.530^af^	11.875 ± 0.459^af^
DIR + Met + Sel	0.955 ± 0.032^c^	30.614 ± 1.759^f^	11.800 ± 0.427^afi^

Values represent the mean of 8 rats ± SEM (one-way ANOVA followed by LSD test).

^a^ *P* < 0.05, ^c^ *P* < 0.001 compared to the control group. ^f^ *P* < 0.001 compared to DIR group. ^i^ *P* < 0.001 compared to DIR + Met group.

**Table 4:** Correlational analysis of the studied parameters.

	Glucose	HOMA-IR index	Adiponectin	GSH	PTP
Glucose (R)	1	0.902**	−0.463**	−0.843**	0.702**
HOMA-IR index (R)	0.902**	1	−0.585**	−0.717**	0.538**
Adiponectin (R)	−0.463**	−0.585**	1	0.434**	−0.515**
GSH (R)	−0.843**	−0.717**	0.434**	1	−0.756**
PTP (R)	0.702**	0.538**	−0.515**	−0.756**	1

(R) Pearson correlation.

**Correlation is significant at *P* < 0.01 level (2-tailed).

## References

[1] Roglic G, Unwin N (2010). Mortality attributable to diabetes: estimates for the year 2010. *Diabetes Research and Clinical Practice*.

[3] Schinner S, Scherbaum WA, Bornstein SR, Barthel A (2005). Molecular mechanisms of insulin resistance. *Diabetic Medicine*.

[4] Basciano H, Federico L, Adeli K (2005). Fructose, insulin resistance, and metabolic dyslipidemia. *Nutrition and Metabolism*.

[5] Kim YD, Park K-G, Lee Y-S (2008). Metformin inhibits hepatic gluconeogenesis through AMP-activated protein kinase-dependent regulation of the orphan nuclear receptor SHP. *Diabetes*.

[6] Wiernsperger NF, Bailey CJ (1999). The antihyperglycaemic effect of metf ormin therapeutic and cellular mechanisms. *Drugs*.

[7] Zhou G, Myers R, Li Y (2001). Role of AMP-activated protein kinase in mechanism of metformin action. *Journal of Clinical Investigation*.

[8] McNeill JH, Delgatty HLM, Battell ML (1991). Insulinlike effects of sodium selenate in streptozocin-induced diabetic rats. *Diabetes*.

[9] Becker DJ, Reul B, Ozcelikay AT, Buchet JP, Henquin J-C, Brichard SM (1996). Oral selenate improves glucose homeostasis and partly reverses abnormal expression of liver glycolytic and gluconeogenic enzymes in diabetic rats. *Diabetologia*.

[10] Kenner KA, Anyanwu E, Olefsky JM, Kusari J (1996). Protein-tyrosine phosphatase 1B is a negative regulator of insulin- and insulin-like growth factor-I-stimulated signaling. *Journal of Biological Chemistry*.

[11] Mueller AS, Pallauf J (2006). Compendium of the antidiabetic effects of supranutritional selenate doses. In vivo and in vitro investigations with type II diabetic db/db mice. *Journal of Nutritional Biochemistry*.

[12] Srinivasan K, Viswanad B, Asrat L, Kaul CL, Ramarao P (2005). Combination of high-fat diet-fed and low-dose streptozotocin-treated rat: a model for type 2 diabetes and pharmacological screening. *Pharmacological Research*.

[13] Matthaei S, Reibold JP, Hamann A (1993). In vivo metformin treatment ameliorates insulin resistance: evidence for potentiation of insulin-induced translocation and increased functional activity of glucose transporters in obese (fa/fa) Zucker rat adipocytes. *Endocrinology*.

[14] Berg EA, Wu JY, Campbell L, Kagey M, Stapleton SR (1995). Insulin-like effects of vanadate and selenate on the expression of glucose-6-phosphate dehydrogenase and fatty acid synthase in diabetic rats. *Biochimie*.

[15] Nader MM, Eissa LA, Gamil NM, Ammar E-SM (2007). Effect of nitric oxide, vitamin E and selenium on streptozotocin induced diabetic rats. *Saudi Pharmaceutical Journal*.

[16] Psyrogiannis A, Kyriazopoulou V, Symeonidis A, Leotsinidis M, Vagenakis AG (2003). Relative iron, ‘overload: in offspring of patients with type 2 diabetes mellitus: a new component in the conundrum of insulin resistance syndrome?’. *Hormones(Athens)*.

[17] Trinder P (1969). Determination of blood glucose using 4-amino phenazone as oxygen acceptor. *Journal of Clinical Pathology*.

[18] Morgan CR, Lazarow A (1962). Immunoassay of insulin using a two-antibody system. *Proceedings of the Society for Experimental Biology and Medicine*.

[19] Matthews DR, Hosker JP, Rudenski AS (1985). Homeostasis model assessment: insulin resistance and *β*-cell function from fasting plasma glucose and insulin concentrations in man. *Diabetologia*.

[20] Allain CC, Poon LS, Chan CSG (1974). Enzymatic determination of total serum cholesterol. *Clinical Chemistry*.

[21] Wahlefeld AW, Bergmeyer HU (1974). Triglycerides determination after enzymatic hydrolysis. *Methods of Enzymatic Analysis*.

[22] Matsubara C, Nishikawa Y, Yoshida Y, Takamura K (1983). A spectrophotometric method for the determination of free fatty acid in serum using acyl-coenzyme A synthetase and acyl-coenzyme A oxidase. *Analytical Biochemistry*.

[23] Nakano Y, Tajima S, Yoshimi A (2006). A novel enzyme-linked immunosorbent assay specific for high-molecular- weight adiponectin. *Journal of Lipid Research*.

[24] Lowry OH, Rosebrough NJ, Farr AL, Randall RJ (1951). Protein measurement with the Folin phenol reagent. *The Journal of Biological Chemistry*.

[25] Beutler E, Duron O, Kelly BM (1963). Improved method for the determination of blood glutathione. *The Journal of Laboratory and Clinical Medicine*.

[26] Lanzetta PA, Alvarez LJ, Reinach PS, Candia OA (1979). An improved assay for nanomole amounts of inorganic phosphate. *Analytical Biochemistry*.

[27] Sözmen EY, Sözmen B, Delen Y, Onat T (2001). Catalase/superoxide dismutase (SOD) and catalase/paraoxonase (PON) ratios may implicate poor glycemic control. *Archives of Medical Research*.

[28] Erejuwa OO, Sulaiman SA, Wahab MSA, Sirajudeen KNS, Salleh MSM, Gurtu S (2011). Glibenclamide or metformin combined with honey improves glycemic control in streptozotocin-induced diabetic rats. *International Journal of Biological Sciences*.

[29] Bansal P, Paul P, Mudgal J (2012). Antidiabetic, antihyperlipidemic and antioxidant effects of the flavonoid rich fraction of *Pilea microphylla* (L.) in high fat diet/streptozotocin-induced diabetes in mice. *Experimental and Toxicologic Pathology*.

[30] Matafome P, Louro T, Rodrigues L (2011). Metformin and atorvastatin combination further protect the liver in type 2 diabetes with hyperlipidaemia. *Diabetes/Metabolism Research and Reviews*.

[31] Hu F, Li X, Zhao L, Feng S, Wang C (2010). Antidiabetic properties of purified polysaccharide from Hedysarum polybotrys. *Canadian Journal of Physiology and Pharmacology*.

[32] Da Silva Xavier G, Leclerc I, Varadi A, Tsuboi T, Moule SK, Rutter GA (2003). Role for AMP-activated protein kinase in glucose-stimulated insulin secretion and preproinsulin gene expression. *Biochemical Journal*.

[33] Zhang S, Kim K-H (1995). Glucose activation of acetyl-CoA carboxylase in association with insulin secretion in a pancreatic *β*-cell line. *Journal of Endocrinology*.

[34] Haddad PS, Benhaddou-Andaloussi A, Martineau L (2011). The in vivo antidiabetic activity of Nigella sativa is mediated through activation of the AMPK pathway and increased muscle Glut4 content. *Evidence-Based Complementary and Alternative Medicine*.

[35] Ong KW, Hsu A, Song L, Huang D, Tan BKH (2011). Polyphenols-rich Vernonia amygdalina shows anti-diabetic effects in streptozotocin-induced diabetic rats. *Journal of Ethnopharmacology*.

[36] Behera GB, Kurnool AN, Baidya M, Kumar BS, Bilal S (2011). Anti-hyperglyceamic, anti-hyperlipidemic and antioxidant activity of the stem of Glinus Oppositifolius (L.) AUG. DC. *International Journal of Pharmacy and Pharmaceutical Sciences*.

[37] Zheng X-K, Li Y-J, Zhang L, Feng W-S, Zhang X (2011). Antihyperglycemic activity of *Selaginella tamariscina* (Beauv.) Spring. *Journal of Ethnopharmacology*.

[38] Metais C, Forcheron F, Abdallah P (2008). Adiponectin receptors: expression in Zucker diabetic rats and effects of fenofibrate and metformin. *Metabolism*.

[39] Alhaider AA, Korashy HM, Sayed-Ahmed MM, Mobark M, Kfoury H, Mansour MA (2011). Metformin attenuates streptozotocin-induced diabetic nephropathy in rats through modulation of oxidative stress genes expression. *Chemico-Biological Interactions*.

[40] Holland W, Morrison T, Chang Y, Wiernsperger N, Stith BJ (2004). Metformin (Glucophage) inhibits tyrosine phosphatase activity to stimulate the insulin receptor tyrosine kinase. *Biochemical Pharmacology*.

[41] Kannappan S, Anuradha CV (2009). Insulin sensitizing actions of fenugreek seed polyphenols, quercetin & metformin in a rat model. *Indian Journal of Medical Research*.

[42] Roy S, Dontamalla SK, Mondru AK, Sannigrahi S, Veerareddy PR (2011). Downregulation of apoptosis and modulation of TGF-*β*1 by sodium selenate prevents streptozotocin-induced diabetic rat renal impairment. *Biological Trace Element Research*.

[43] Aydemir-Koksoy A, Bilginoglu A, Sariahmetoglu M, Schulz R, Turan B (2010). Antioxidant treatment protects diabetic rats from cardiac dysfunction by preserving contractile protein targets of oxidative stress. *Journal of Nutritional Biochemistry*.

[44] Bloch-Damti A, Bashan N (2005). Proposed mechanisms for the induction of insulin resistance by oxidative stress. *Antioxidants and Redox Signaling*.

[45] Mueller AS, Pallauf J, Rafael J (2003). The chemical form of selenium affects insulinomimetic properties of the trace element: investigations in type II diabetic dbdb mice. *Journal of Nutritional Biochemistry*.

[46] Iizuka Y, Ueda Y, Yagi Y, Sakurai E (2010). Significant improvement of insulin resistance of GK rats by treatment with sodium selenate. *Biological Trace Element Research*.

[47] Bayraktutan U (2002). Free radicals, diabetes and endothelial dysfunction. *Diabetes, Obesity and Metabolism*.

[48] Bakopanos E, Silva JE (2000). Thiazolidinediones inhibit the expression of *β*3-adrenergic receptors at a transcriptional level. *Diabetes*.

[49] Szalkowski D, White-Carrington S, Berger J, Zhang B (1995). Antidiabetic thiazolidinediones block the inhibitory effect of tumor necrosis factor-*α* on differentiation, insulin-stimulated glucose uptake, and gene expression in 3T3-L1 cells. *Endocrinology*.

[50] Kintscher U, Unger T (2005). Vascular protection in diabetes: a pharmacological view of angiotensin II type 1 receptor blockers. *Acta Diabetologica*.

[51] Brigelius-Flohé R (1999). Tissue-specific functions of individual glutathione peroxidases. *Free Radical Biology and Medicine*.

[52] Müller AS, Most E, Pallauf J (2005). Effects of a supranutritional dose of selenate compared with selenite on insulin sensitivity in type II diabetic dbdb mice. *Journal of Animal Physiology and Animal Nutrition*.

